# Shone Complex: A Case Report of Congenital Heart Disease Detected Using Point-of-care Ultrasound

**DOI:** 10.5811/cpcem.1319

**Published:** 2023-08-09

**Authors:** Jordan C. Seaback, David A. Masneri, Jacob H. Schoeneck

**Affiliations:** Atrium Health Wake Forest Baptist, Department of Emergency Medicine, Winston Salem, North Carolina

**Keywords:** case report, Shone complex, point-of-care ultrasound, cardiogenic shock, congenital heart disease

## Abstract

**Introduction:**

Undiagnosed congenital heart disease and management of pediatric cardiogenic shock presents a diagnostic challenge for the emergency clinician. These diagnoses are rare and require a high index of suspicion given the overlap with more common pediatric pathology. Point-of-care ultrasound can assist in differentiating these presentations. We present a case of neonatal cardiogenic shock secondary to a previously undiagnosed congenital heart disease, specifically Shone complex, detected using point-of-care ultrasound.

**Case Report:**

A six-week-old female presented with severe respiratory distress and was found to be in cardiogenic shock secondary to a previously undiagnosed congenital heart defect.

**Conclusion:**

Initial diagnosis of congenital heart disease is uncommon in the emergency department, but it should be recognized by clinicians given the high associated morbidity and mortality. Point-of-care ultrasound is a powerful tool to assist in evaluating for cardiac abnormalities as an etiology for undifferentiated shock in the pediatric population.

## INTRODUCTION

Congenital anomalies are the leading cause of infant mortality in the United States with congenital heart disease accounting for 30–50% of these deaths.^1^ Despite advances in prenatal ultrasound screening, detection of congenital heart disease is missed in approximately half of cases. National screening programs in several developed countries have reported detection rates of only 30–60%.^2^ While ED presentation of an undiagnosed congenital heart disease is rare, this diagnosis must be considered. Patient presentations can vary substantially and include nonspecific complaints such as respiratory distress, lethargy, failure to thrive and, rarely, cardiogenic shock.^3^

Early use of point-of-care ultrasound in this patient population can provide invaluable information for the emergency clinician. Our case highlights the utility of point-of-care ultrasound to identify a previously undiagnosed congenital heart defect in a neonate presenting in cardiogenic shock. Specifically, our case highlights Shone complex, a rare congenital heart disease comprised of four left-sided heart defects including a parachute mitral valve, subaortic stenosis, a supravalvular mitral ring, and coarctation of the aorta.^4^

## CASE REPORT

A six-week-old female presented to our ED via ambulance from her pediatrician’s office for respiratory distress. She had no known past medical history and was born at 38 weeks via induced vaginal delivery with routine prenatal care. Her mother described a history of increased work of breathing, wheezing, and grunting respirations since birth, which had noticeably worsened in the prior week. The patient had mild associated congestion and rhinorrhea, but her mother denied other infectious symptoms. At the pediatrician’s office, the patient was found to have an oxygen saturation of 70% and was placed on a non-rebreather with minimal improvement.

Her initial vital signs on arrival to the ED showed hypothermia with a temperature of 35.2° Celsius, a tachycardic heart rate of 169 beats per minute, tachypnea with a respiratory rate of 50 breaths per minute, and persistent hypoxia at 68% despite oxygenation by non-rebreather. Obtaining an accurate blood pressure was challenging, but the highest measurement was 38/23 millimeters of mercury. Physical examination was notable for an ill-appearing, mottled, neonatal female. Her heart and lung sounds were diminished on auscultation, with diffuse rales that were more prominent in the right lung. She was noted to have absent femoral pulses.

**Image 1:**
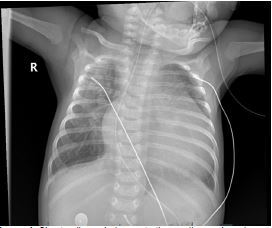
Chest radiograph demonstrating cardiomegaly and interstitial prominence suggestive of pulmonary edema.

Given the patient’s poor oxygen saturation and increased work of breathing, she was transitioned to high-flow nasal cannula. A chest radiograph was obtained and demonstrated cardiomegaly with pulmonary interstitial edema (Image 1).

**Image 2:**
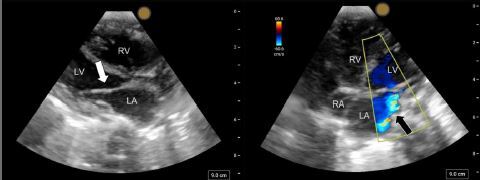
Parasternal long axis (left) depicting restricted movement and abnormal morphology of the anterior mitral valve leaflet (white arrow). Apical four chamber (right) with visualization of the left atrium (LA), left ventricle (LV), right atrium (RA), and right ventricle (RV) with a mitral regurgitation jet on color flow Doppler (black arrow).

Focused point-of-care echocardiography was performed, which showed poor left ventricular contractility with an ejection fraction visually estimated to be less than 15%, restricted motion of the mitral valve leaflets with abnormal valve morphology, and a mitral regurgitation jet (Image 2 and Video). These findings raised the concern for cardiogenic shock secondary to a previously undiagnosed congenital heart defect. An epinephrine infusion was started to provide inotropic support with improvement in the patient’s blood pressure.

**CPC-EM Capsule**

What do we already know about this clinical entity?

*Undiagnosed congenital heart disease can be a diagnostic challenge given the overlap with more common pediatric pathology.*

What makes this presentation of disease reportable?

*Cardiogenic shock from an undiagnosed congenital heart disease such as Shone complex is a rare but important consideration in the undifferentiated sick neonate.*

What is the major learning point?

*Point-of-care ultrasound is a powerful tool to assist in evaluating for cardiac abnormalities as an etiology for undifferentiated shock in the pediatric population.*

How might this improve emergency medicine practice?

*Point-of-care ultrasound in the sick pediatric patient can provide real-time diagnostic information and facilitate early collaboration with consultants.*

Ultimately her oxygenation and work of breathing failed to improve on high-flow nasal cannula, and she was intubated. Fentanyl was given for induction and sedation, but paralytics were avoided to ensure preservation of her respiratory drive.

Cardiology was consulted and a complete echocardiogram was performed, which confirmed the diagnosis of congenital heart disease, more specifically an incomplete Shone complex. We considered initiation of alprostadil in case of a ductal-dependent lesion, and this was eventually administered after transfer to the pediatric intensive care unit. Her clinical course was complicated by significant end-organ damage including acute renal failure requiring continuous renal replacement therapy and seizures. She underwent coarctation repair several days later, which she tolerated well. Her ejection fraction, renal failure, and seizures improved, and she was discharged home one month after admission.

## DISCUSSION

Shone complex is a rare congenital heart disease comprising four left-sided heart defects including a parachute mitral valve, subaortic stenosis, a supravalvular mitral ring, and coarctation of the aorta. It is estimated that Shone complex comprises 0.6–0.7% of all congenital heart disease.^5^–^7^ If all four defects are present, the complex is termed “complete,” although the majority of cases have three or fewer defects present and are considered “incomplete.”^4^,^5^ Several other congenital heart defects are frequently associated with Shone complex including bicuspid aortic valve, atrial and ventricular septal defects, patent ductus arteriosus, and interrupted aortic arch.^7^ The degree of mitral valve obstruction in Shone complex correlates with a poor prognosis and earlier onset of symptoms. Symptoms can be non-specific including dyspnea, cough, failure to thrive, poor feeding, lethargy, wheezing, and recurrent respiratory tract infections. Long-term prognosis is generally poor with many patients requiring multiple surgeries and ultimately heart transplantation.^8^

While the definitive diagnosis of Shone complex is well outside the scope of point-of-care ultrasound, some defining sonographic abnormalities can be striking and readily appreciated. Our point-of-care echocardiography was performed using the phased array probe placed in the standard positioning. There are no differences in probe placement between the adult and pediatric populations. The “parachute” shape of the mitral valve has a distinct sonographic appearance and may markedly restrict the movement of the valve leaflets, which is easily appreciated on dynamic imaging (Image 2 and Video). Mitral insufficiency and regurgitation can be visualized with color Doppler in the apical four-chamber view. The diagnosis of subaortic stenosis is more complicated requiring measurement of post-stenotic flow velocities. Views of the aortic arch and descending aorta can be obtained with the probe placed in the suprasternal notch, although the diagnosis of aortic coarctation is challenging and requires excellent visualization for accurate measurements and Doppler evaluation.^7^

In our case, a previously healthy six-week-old with reported routine prenatal care presented in decompensated cardiogenic shock. She was ultimately found to have an incomplete form of Shone complex with a bicuspid aortic valve, a parachute mitral valve, and critical postductal coarctation of the aorta. She did not have subaortic stenosis or a supravalvular mitral ring. Her case proved to be a diagnostic challenge as we began with a working diagnosis of bronchiolitis, prompting the initiation of high-flow nasal cannula. We additionally considered sepsis, toxin-mediated, congenital metabolic disorders, and non-accidental trauma as other etiologies for her decompensation.

The cardiomegaly and interstitial prominence on her chest radiograph initially raised our concern for underlying heart failure. However, the chest radiograph alone did not provide enough diagnostic information for proper management. In the acute setting, the anatomic, physiological, and hemodynamic information that functional echocardiography provides can be used in targeting specific interventions and evaluating response to treatment.^9^ Point-of-care ultrasound enabled us to assess for additional causes of cardiomegaly, including pericardial effusions and cardiac tamponade, which would have substantially changed our management. Ultimately, POCUS revealed severe cardiac anomalies that facilitated early consultation and collaboration with our pediatric cardiologists.

In general, pediatric cardiogenic shock with left ventricular systolic failure, such as in Shone complex, is managed with inotropes to increase contractility and reduce afterload. Diuretics may also be used to decrease both pulmonary vascular congestion and preload.^10^ Diuretics were not indicated in the acute management of our patient in extremis and were not recommended by our cardiology colleagues. Clinicians may also consider prostaglandins such as alprostadil in the case of a ductal-dependent lesion. However, this should only be given in conjunction with cardiology consultation, as side effects of this medication can be profound and include worsening hypotension and apnea.^11^

The decision to intubate congenital heart patients is debated given the risk of decreasing preload and worsening cardiac output. In severe cases, intubation and positive-pressure ventilation may assist in decreasing left ventricular transmural pressure and afterload as well as the metabolic demands of respiratory muscles. Our decision to proceed with intubation was made in conjunction with our pediatric intensivists and cardiology colleagues as sedation and intubation can cause rapid deterioration in which mechanical cardiac support may be needed.^10^ Prior to intubation we started an epinephrine infusion for inotropic support and used fentanyl only for sedation without paralytics to ensure preservation of the patient’s respiratory drive.

## CONCLUSION

Delayed diagnosis of congenital heart disease is rare but carries a high risk of morbidity and mortality. These cases can be diagnostic challenges given that heart failure and cardiogenic shock is rare in the pediatric and neonatal population. Symptoms can also overlap with more common conditions including bronchiolitis or pneumonia. Emergency physicians should be aware of these lesions and the role of point-of-care ultrasound to assist with diagnosis.

## Supplementary Information

Video Narrated video describing the chest radiograph with cardiomegaly and pulmonary interstitial edema. Video clip of the point-of-care parasternal long-axis view demonstrates abnormal mitral valve morphology with tethering and restricted motion of the mitral valve leaflet (white arrow). Point-of-care apical four-chamber view demonstrates color flow Doppler with a mitral regurgitant jet (white arrow). In the final clips, a cardiology-performed echocardiogram in the parasternal long-axis and short-axis views reveal a “parachute” mitral valve (white arrow) and bicuspid aortic valve (white arrow), respectively. The suprasternal aortic view reveals coarctation of the aorta (white arrow).
